# Common DNA Markers Can Account for More Than Half of the Genetic Influence on Cognitive Abilities

**DOI:** 10.1177/0956797612457952

**Published:** 2013-04

**Authors:** Robert Plomin, Claire M. A. Haworth, Emma L. Meaburn, Thomas S. Price, Wellcome Trust Case Control Consortium 2, Oliver S. P. Davis

**Affiliations:** 1 MRC Social, Genetic & Developmental Psychiatry Centre, Institute of Psychiatry, King’s College London; 2 Department of Psychological Sciences, Birkbeck, University of London; 3 Institute for Translational Medicine and Therapeutics, University of Pennsylvania School of Medicine; 4 UCL Genetics Institute, Research Department of Genetics, Evolution and Environment, University College London

**Keywords:** cognitive ability, behavioral genetics, cognitive development, genetics

## Abstract

For nearly a century, twin and adoption studies have yielded substantial estimates of heritability for cognitive abilities, although it has proved difficult for genomewide-association studies to identify the genetic variants that account for this heritability (i.e., the missing-heritability problem). However, a new approach, genomewide complex-trait analysis (GCTA), forgoes the identification of individual variants to estimate the total heritability captured by common DNA markers on genotyping arrays. In the same sample of 3,154 pairs of 12-year-old twins, we directly compared twin-study heritability estimates for cognitive abilities (language, verbal, nonverbal, and general) with GCTA estimates captured by 1.7 million DNA markers. We found that DNA markers tagged by the array accounted for .66 of the estimated heritability, reaffirming that cognitive abilities are heritable. Larger sample sizes alone will be sufficient to identify many of the genetic variants that influence cognitive abilities.

Cognitive abilities predict educational attainment, income, health, and longevity, and thus contribute importantly to the intellectual capital of knowledge-based societies (Deary, 2012). Since the 1920s, twin and adoption studies have investigated the genetic and environmental origins of individual differences in cognitive abilities; scores of such studies have consistently yielded estimates of substantial heritability (i.e., the extent to which genetic variance can account for observed, or phenotypic, variance; Deary, Johnson, & Houlihan, 2009). Meta-analyses of these studies have yielded heritability estimates of about .50 for general cognitive ability, the most well-studied cognitive trait (Plomin, DeFries, Knopik, & Neiderhiser, 2013).

Although the consensus concerning heritability of cognitive abilities is not unanimous (Nisbett et al., 2012), twin and adoption studies have moved beyond asking whether and how much genes influence cognitive abilities to asking how they do so (Haworth & Plomin, 2010). For example, investigating how genetic influence on cognitive abilities develops has revealed a steady increase in the heritability of general cognitive ability from childhood through adulthood (Haworth et al., 2010). Another important developmental finding is that genes contribute primarily to stability from age to age, although some new genetic effects come into play during the major cognitive transformations from early to middle childhood (Davis, Haworth, & Plomin, 2009a) and from middle childhood to adolescence (van Soelen et al., 2011). Another active area of research focuses on genetic links between cognitive abilities and brain structure and function (Deary, Penke, & Johnson, 2010).

Few discoveries would have greater impact than identifying some of the genes responsible for the heritability of cognitive abilities. The first attempts to find genes associated with cognitive abilities focused on genes involved in brain function (Payton, 2009). However, attempts to replicate reported associations between such candidate genes and cognitive abilities have often failed (Chabris et al., 2012). During the past few years, gene hunting has been revolutionized by an atheoretical approach known as genomewide association (GWA; Plomin, 2012). GWA studies assess associations between a trait and hundreds of thousands of DNA markers (typically single-nucleotide polymorphisms, SNPs) distributed throughout the 3 billion nucleotide bases of the genome genotyped simultaneously using a DNA array the size of a postage stamp (McCarthy et al., 2008). In the past 5 years, nearly 1,500 GWA studies have identified more than 200 associations between SNPs and complex (i.e., not single-gene) traits, mostly common medical disorders (Hindorff et al., 2013; Visscher, Brown, McCarthy, & Yang, 2012).

If all the genes responsible for the heritability of traits could be identified, there would no longer be any need for twin or adoption studies. However, there is a great gap between genes identified so far in GWA studies and heritability estimates—the *missing-heritability problem* (Maher, 2008). One of the most far-reaching results of GWA studies is to show that there are no genes of large effect size in the population, which means that the heritability of complex traits is probably due to many genes of small effect size, and this means that associations will be difficult to detect and replicate (Plomin, 2012). For example, the first GWA studies of general cognitive ability (Davies et al., 2011; Davis et al., 2010) were powered to detect associations that account for as little as .01 of the variance, but they came up empty-handed because the associations with the largest effect accounted for less than .005 of the variance. One of many possible reasons for the missing-heritability problem is that the common SNPs (i.e., SNPs for which the frequency of the less frequent allele is greater than .01) incorporated in commercially available DNA arrays miss the contribution of rare DNA variants (Cirulli & Goldstein, 2010). Another possibility is that heritability has been overestimated by twin and adoption studies.

## Genomewide Complex-Trait Analysis

The study reported here addressed both of these possibilities by comparing twin-based estimates of heritability for cognitive abilities with estimates from a new method that is population based rather than family based. The method, called *genomewide complex-trait analysis* (GCTA), can be used to estimate genetic variance accounted for by all the SNPs that have been genotyped in any sample, not just samples consisting of special family members such as twins or adoptees (Lee, Wray, Goddard, & Visscher, 2011; Yang, Lee, Goddard, & Visscher, 2011; Yang, Manolio, et al., 2011). However, GCTA requires large samples in which each individual has been genotyped for hundreds of thousands of DNA markers, typically SNPs. Although these requirements might seem daunting, they are also the requirements for GWA, which means that the data from many GWA studies, including GWA studies of cognitive abilities, can be used to conduct GCTA.

GCTA does not identify specific genes associated with traits. Instead, it uses chance similarity across hundreds of thousands of SNPs to predict phenotypic similarity pair by pair in a large sample of unrelated individuals. The essence of GCTA is to estimate genetic influence on a trait by predicting phenotypic similarity for each pair of individuals in the sample from their total SNP similarity. In contrast to the twin method, which estimates heritability by comparing phenotypic similarity of identical and fraternal twin pairs, whose genetic similarity is roughly 1.00 and .50, respectively, GCTA relies on comparisons of pairs of individuals whose genetic similarity varies from .00 to .02. GCTA extracts this tiny genetic signal from the noise of hundreds of thousands of SNPs using the massive information available from a matrix of thousands of individuals, each compared pair by pair with every other individual in the sample; for example, the 3,000-plus individuals in the present sample provided nearly 5 million pairwise comparisons.

GCTA genetic similarity is not limited to the genotyped SNPs themselves, but also includes unknown causal variants to the extent that they are correlated with the SNPs. Mendel’s second law of inheritance is that genes (as they are now called) are inherited independently (a phenomenon now called linkage equilibrium), but Mendel did not know that genes can be on the same chromosome, in which case they are not inherited independently (linkage disequilibrium). This violation of Mendel’s second law is complicated by the fact that during meiosis, on average each pair of chromosomes—one from the mother and one from the father—crosses over (recombines) once; in the population, genes on the same chromosome are separated by this process of recombination to the extent that they are not close together on the chromosome. GCTA provides a lower-limit estimate of heritability because it misses genetic influence due to causal variants that are not highly correlated with the common SNPs on genotyping arrays.

A difference between GCTA estimates and twin-study estimates of heritability is that GCTA estimates only additive genetic effects, whereas the twin method captures nonadditive as well as additive genetic effects. Additive genetic effects are caused by the independent effects of alleles, which add up in their effect on a trait; nonadditive genetic effects are those that interact. Because GCTA adds up the effect of each SNP, it does not include gene-gene interaction effects; the twin method captures nonadditive as well as additive genetic effects because the DNA sequence of identical twins is virtually identical and thus they share all genetic effects, including nonadditive ones (see Plomin et al., 2013, for details).

GCTA has been used to estimate heritability as captured by genotyping arrays for height (Yang et al., 2010), weight (Yang, Manolio, et al., 2011), psychiatric and other medical disorders (Lee et al., 2012; Lee et al., 2011; Lubke et al., 2012), and personality (Vinkhuyzen, Pedersen, et al., 2012). GCTA was first applied to cognitive ability in a study of 3,500 unrelated adults, which yielded heritability estimates of .40 and .51 for crystallized and fluid intelligence, respectively (Davies et al., 2011). The GCTA estimate for general cognitive ability was .47 in a meta-analysis across three studies involving nearly 10,000 adults (Chabris et al., 2012) and .48 in a study of nearly 2 thousand 11-year-old children (Deary et al., 2012).

The GCTA results from these initial studies appear to account for a substantial portion of the heritability of general cognitive ability found in twin studies, which, as mentioned earlier, meta-analyses have found to be about .50. However, the extent to which GCTA estimates for cognitive abilities account for family-based estimates deserves closer investigation for three reasons. First, as already mentioned, GCTA estimates depend on extracting a tiny signal from much noise and thus entail large standard errors (e.g., .11 in the study by Davies et al., 2011, which included 3,500 individuals). Second, because twin-based heritability estimates vary by sample, age, and measure, comparisons with GCTA estimates should not rely solely on averaged estimates of heritability from the world’s literature on general cognitive ability. In addition, for cognitive abilities other than general cognitive ability, meta-analytic estimates of heritability are not available.

The third reason that there is a need for greater precision in determining the extent to which GCTA-based estimates for cognitive abilities account for twin-based heritability estimates is that GCTA estimates provide a crucial clue for solving the missing-heritability problem. As mentioned earlier, one possible explanation of the missing heritability is that rare genetic variants have not been considered in addition to the common SNPs that are detected by available DNA arrays. However, to the extent that GCTA estimates that rely on common SNPs can account for heritability estimates from twin studies, one can conclude that common SNPs alone can predict cognitive abilities if sample sizes are sufficiently large. This would mean that, with sample sizes in the hundreds of thousands, as in research on height (Lango Allen et al., 2010) and weight (Speliotes et al., 2010), many replicable associations between DNA and cognitive abilities could be found.

The purpose of the present study was to compare GCTA estimates of the heritability of cognitive abilities with heritability estimates obtained with the classical twin design—using the same sample assessed at the same age with the same measures of diverse cognitive abilities, not just general cognitive ability. The sample included 3,154 pairs of 12-year-old twins; one member of each pair had been genotyped on the Affymetrix 6.0 GeneChip (Affymetrix, Santa Clara, CA). We investigated verbal and nonverbal cognitive abilities and language ability, in addition to general cognitive ability and the anchor variables of height and weight.

## Method

This Method section is brief because descriptions of the sample and measures have been published previously (Davis, Haworth, & Plomin, 2009b). The sample was from the Twins Early Development Study (TEDS; Oliver & Plomin, 2007), a representative sample of families in the United Kingdom (Kovas, Haworth, Dale, & Plomin, 2007). Cognitive data were available for 5,434 pairs at age 12 (Davis et al., 2009b); however, the twin analyses presented here, although very similar to those reported by Davis et al. (2009b), were based on only the 3,154 pairs of twins that included a member for whom GWA genotyping data were available. Restricting the sample in this way provided an even better comparison with the GCTA estimates.

Details of the measures are described in Davis et al. (2009b): Composite scores were created for language ability (three tests), verbal cognitive ability (two tests), nonverbal cognitive ability (two tests), and general cognitive ability (verbal + nonverbal), all of which were assessed via Web-based testing (Haworth et al., 2007). Heritability was estimated from our twin data using standard model fitting, as described in Davis et al. (2009b).

Genotyping on the Affymetrix 6.0 GeneChip and subsequent quality control were carried out as part of the Wellcome Trust Case Control Consortium 2 project (The UK IBD Genetics Consortium & the Wellcome Trust Case Control Consortium 2, 2009) for 3,154 individuals (1 member of each twin pair) for whom cognitive data at age 12 were also available. In addition to nearly 700,000 genotyped SNPs, more than 1 million other SNPs were imputed using IMPUTE Version 2 software (Howie, Donnelly, & Marchini, 2009). GCTA estimates were obtained using the GCTA software package (Yang, Lee, et al., 2011).

## Results

**Fig. 1.**
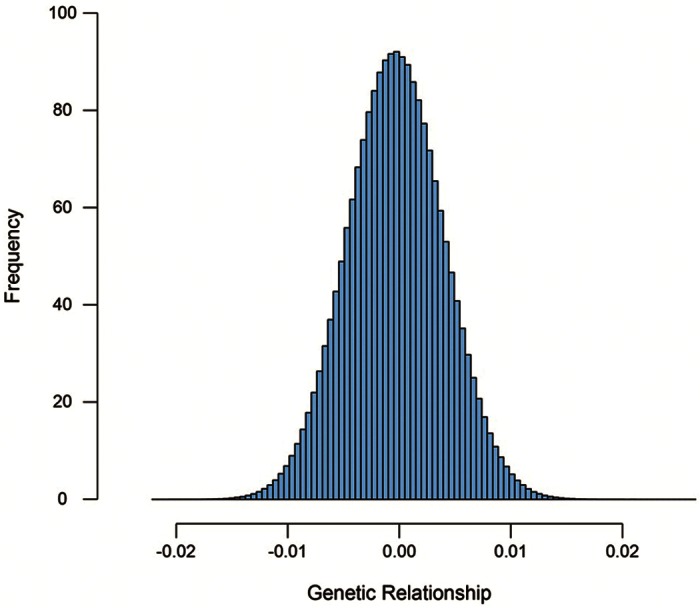
Distribution of chance genetic similarity across 1.7 million single-nucleotide polymorphisms for 3,154 unrelated individuals, pair by pair. The sample was taken from the Twins Early Development Study sample.

Figure 1 shows the present sample’s (3,154 unrelated individuals) normal distribution of chance genetic similarity pair by pair across the 1.7 million genotyped and imputed SNPs, as obtained using the GCTA software package (Yang, Lee, et al., 2011). The figure illustrates the point that more than 90% of the pairings varied no more than 1% from the sample mean. GCTA uses each pair’s total SNP similarity to predict phenotypic similarity pair by pair. Table 1 presents GCTA estimates and confidence intervals for the anchor variables (height and weight) and the four cognitive scores. All GCTA heritability estimates were significant, although their 95% confidence intervals were wide. The GCTA heritability estimates were .35 for height, .42 for weight, and .35 for general cognitive ability. The GCTA heritability estimates for the other cognitive variables ranged from .20 to .29.

**Table 1.** Comparison of Estimates of Heritability Obtained Using Genomewide Complex-Trait Analysis (GCTA) and the Twin Design

Measure	GCTA estimate	Twin-based estimate	Ratio of GCTA estimate to twin-based estimate
Weight	.42 [.19, .65]	.84 [.80, .88]	.50
Height	.35 [.11, .58]	.80 [.76, .84]	.44
General cognitive ability	.35 [.12, .58]	.46 [.42, .52]	.76
Nonverbal cognitive ability	.20 [.01, .43]	.42 [.36, .48]	.48
Verbal cognitive ability	.26 [.04, .49]	.40 [.35, .46]	.65
Language ability	.29 [.06, .53]	.39 [.34, .44]	.74

Note: Numbers inside brackets are 95% confidence intervals.

Twin-based heritability estimates for the same sample, shown in the second column of Table 1, were .80 for height and .84 for weight, and ranged from .39 to .46 for the cognitive variables. The third column in the table indicates the proportion of each twin heritability estimate that is accounted for by the GCTA estimate. The proportions are .44 for height and .50 for weight. For cognitive abilities, the proportions vary from .48, for nonverbal ability, to .76, for general cognitive ability.

## Discussion

This is the first study in which GCTA estimates of heritability for diverse cognitive abilities were compared directly with twin-based estimates using the same measures at the same age in the same sample. The Affymetrix 6.0 DNA array yielded GCTA estimates that accounted on average for .66 of the twin heritability estimates for language, verbal, nonverbal, and general cognitive abilities. Note that the GCTA estimates accounted for a greater proportion of the twin heritability estimates in the case of cognitive abilities than in the case of height (.44) and weight (.50).

Among the cognitive scores, general cognitive ability had the highest GCTA estimate (.35) and the highest ratio (.76) between its GCTA estimate and its twin heritability estimate (.46). Although the GCTA estimates did not differ significantly among the cognitive abilities because of their large confidence intervals (see Table 1), previously reported GCTA estimates for general cognitive ability were also substantial for children (Deary et al., 2012) and adults (Chabris et al., 2012; Davies et al., 2011). If valid, this finding suggests that general cognitive ability is a good candidate for narrowing the missing-heritability gap using the common SNPs on current DNA arrays with much larger samples. This is fortunate because far more GWA data are available for general cognitive ability than for other cognitive abilities.

Why might these common SNPs tag general cognitive ability more than height and weight? Common SNPs are likely to be common because they are old, having spread through the population over many generations, but there seems no obvious reason why the evolutionary architecture for general cognitive ability should differ from height in this way. However, there is one major genetic difference between cognitive and physical traits: Assortative mating (nonrandom mating) is at least twice as great for general cognitive ability (correlation between spouses: ~.45) as for height and weight (~.20; Plomin et al., 2013). The effect of assortative mating is to increase additive genetic variance because children receive correlated genetic influences from their parents, which spreads out the distribution; moreover, the effects of assortative mating accumulate generation after generation. If assortative mating is responsible for the fact that common SNPs tag general cognitive ability more than height and weight, then verbal abilities should show greater GCTA/twin heritability ratios than nonverbal abilities do because verbal abilities show more assortative mating than nonverbal abilities (correlation between spouses: ~.50 vs. .30). The results in Table 1 are consistent with this hypothesis: The GCTA/twin heritability ratio is .65 for verbal ability and .48 for nonverbal ability.

The strongest test of the effect of assortative mating would involve a different application of GCTA: Rather than using GCTA to estimate genetic similarity between pairs of unrelated individuals, researchers could use GCTA to estimate genetic similarity between spouses. This GCTA index of assortative mating could then be related to traits to assess the contribution of assortative mating. In the present case, the prediction is that the GCTA index of assortative mating will be associated more strongly with cognitive than with physical traits and more strongly with verbal than with nonverbal abilities. However, we were unable to test this hypothesis in the present sample because we did not have DNA from the parents of the twins.

These results suggest that research using current DNA arrays with their common SNPs could identify genes that account for about two thirds of the heritability of cognitive abilities simply by including larger samples. But why is the cup only two-thirds full? Accounting for the rest of the missing heritability is likely to require other DNA variants not well tagged by the common SNPs on current DNA arrays (Gibson, 2012). Although such data are not currently available, this situation will eventually be resolved by whole-genome sequencing data (Plomin, 2012). Until then, researchers need to consider the possibility that twin heritability estimates are inflated. One argument against this possibility is that twin-based heritability estimates for cognitive abilities are in line with estimates from adoption studies and family studies, even though the adoption and family designs have different assumptions than the twin design does (Plomin et al., 2013). A specific reason why GCTA heritability estimates might be lower than twin-based estimates was mentioned earlier: GCTA estimates only additive genetic effects, whereas twin estimates include nonadditive as well as additive effects of genes. Although twin-based estimates of heritability for general cognitive ability support additive genetic models, some evidence for nonadditive genetic effects is found when assortative mating, which is substantial for cognitive abilities, is taken into account (Vinkhuyzen, van der Sluis, Maes, & Posthuma, 2012).

Although GCTA requires very large samples genotyped on very large numbers of DNA markers, it is a welcome addition to the armamentarium of quantitative genetics because it is such a different approach—based on DNA markers in the population, rather than on family relationships—and can be used in any large sample of unrelated individuals rather than requiring special family members, such as twins. GCTA can be used to confirm any findings that have emerged from quantitative genetic research. For example, the heritability of general cognitive ability increases from childhood through adulthood (Haworth et al., 2010); GCTA estimates would also be expected to increase across development.

GCTA can be extended to multivariate analyses that address the genetic covariance between traits. The first multivariate GCTA analysis yielded a genetic correlation of .62 in a 50-year longitudinal study of general cognitive ability from childhood to old age (Deary et al., 2012). This confirms family-based genetic research on cognitive abilities showing that genes largely account for age-to-age stability. Multivariate GCTA models can also be used to test other hypotheses that have emerged from family-based genetic research on cognitive abilities, such as the generalist-genes hypothesis, which posits high genetic correlations among diverse cognitive abilities and disabilities (Plomin & Kovas, 2005). Although GCTA analysis and other DNA-based methods are exciting additions to behavioral genetic research, we suggest that traditional quantitative-genetic methods, such as twin and adoption studies, will continue to make important contributions to understanding how genotypes become phenotypes, in part because twin and adoption studies are as much studies of environmental influence as they are of genetic influence (Haworth & Plomin, 2010).

In summary, GCTA estimates confirmed about two thirds of twin-study estimates of heritability for cognitive abilities, using the same measures at the same age in the same sample. This finding implies that, with sufficiently large sample sizes, many genes associated with cognitive abilities can be identified using the common SNPs on current DNA arrays. Whole-genome sequencing might help to close the rest of the missing-heritability gap by identifying rare DNA variants that contribute to the heritability of cognitive abilities, although other possibilities remain, including the possibility that twin and adoption studies have overestimated heritability. GCTA might also mark the beginning of the end of the nature-nurture controversy because it is much more difficult to dispute DNA-based evidence for genetic influence than it is to question the results of twin and adoption studies. Nonetheless, the ultimate goal is to find the specific DNA sequences responsible for the widespread influence of genetics on individual differences in behavior—nucleotides G, C, T, and A, rather than GCTA.
